# Full Genome of Influenza A (H7N9) Virus Derived by Direct Sequencing without Culture

**DOI:** 10.3201/eid1911.130664

**Published:** 2013-11

**Authors:** Xianwen Ren, Fan Yang, Yongfeng Hu, Ting Zhang, Liguo Liu, Jie Dong, Lilian Sun, Yafang Zhu, Yan Xiao, Li Li, Jian Yang, Jianwei Wang, Qi Jin

**Affiliations:** MOH Key Laboratory of Systems Biology of Pathogens, Beijing, China

**Keywords:** H7N9, influenza A virus, deep sequencing, direct sequencing, culture-free, avian-origin, influenza, viruses

## Abstract

An epidemic caused by influenza A (H7N9) virus was recently reported in China. Deep sequencing revealed the full genome of the virus obtained directly from a patient’s sputum without virus culture. The full genome showed substantial sequence heterogeneity and large differences compared with that from embryonated chicken eggs.

Recently, a novel influenza A (H7N9) virus infected humans in China (*1*,*2*), leading to great concerns about its threat to public health (*3*). However, almost all the current genomes of the novel subtype H7N9 virus have been sequenced after culture in embryonated chicken eggs or mammalian cells. Switching the evolutionary selection pressure from in vivo human respiratory tract to embryonated chicken eggs might introduce mutations into the final genome sequences during culture (*4*). We report determination of the full genome of the influenza A (H7N9) virus derived directly by deep sequencing, without virus culture, from a sputum specimen of an infected human. Deep sequencing provides a direct way to evaluate the genome characteristics and potential virulence and transmissibility of the novel influenza A (H7N9) virus.

## The Study

We collected a sputum specimen from a 54-year-old woman with fever, cough, sputum production, and pneumonia. Influenza A (H7N9) virus was detected in the specimen by specific real-time reverse transcription PCR (RT-PCR). The specimen was then processed with a viral particle–protected nucleic acid purification method (*5*). Total RNA was extracted and amplified by sequence-independent PCR (*5*) and then sequenced with an Illumina/Solexa GAII sequencer (Illumina, San Diego, CA, USA). Reads generated by the Illumina/Solexa GAII with lengths of 80 bases were directly aligned to those nucleotide sequences of influenza A viruses in the National Center for Biotechnology Information nonredundant nucleotide database by the blastn program in the BLAST (*6*) software package, version 2.2.22 (www.ncbi.nlm.nih.gov/blast) with parameters −e 1e−5 −F T (−e 1e−5 for selection of highly similar reads and −F T for masking the low-complexity reads) after filtering of the sequence adapters and RT-PCR primers. No assembly was performed before alignment. We obtained 19,177 reads aligned to influenza A viruses.

We then conducted a reference-guided assembly based on the 19,177 reads by the Seqman program in the DNAStar software package version 7.1 (http://www.dnastar.com). The novel influenza A (H7N9) virus A/Anhui/1/2013was selected as the reference. With 80% minimum sequence similarity tolerance and 12 bp minimum match size, those 19,177 reads were assembled into 439 contigs. The top 8 contigs covered by the most reads corresponded to the 8 genome segments of the novel influenza A (H7N9) virus. The other contigs did not align to the reference virus, which might have resulted from sequencing or assembling errors. Calculating the consensus sequence, we obtained the genome of the influenza A (H7N9) virus directly from the sputum specimen of this patient. Further RT-PCR and Sanger sequencing confirmed the quality of the assembled subtype H7N9virus genome. Sequences were deposited in GenBank under accession nos. KF226105–KF226120 and KF278742–KF278749.

The influenza A (H7N9) genome that we report varies from that obtained by Sanger sequencing after passage in the allantoic sac and amniotic cavity of 9–11-day-old specific pathogen–free embryonated chicken eggs for 48–72 hours at 35°C (Table 1). In the nucleocapsid protein (NP) segment, 15 point mutations were found; 13 were synonymous and 2 induced amino acid changes S321N and M371I. In the nonstructural (NS) protein segment, 5 point mutations were found; all caused amino acid changes R59H, P107L, and V111Q. In the polymerase acidic (PA) protein segment, 3 point mutations were found, 1 of which caused amino acid change V707F. In the polymerase basic 1 (PB1) protein segment, 2 point mutations were found, both of which were synonymous. In the PB2 segment, 2 point mutations were found, 1 of which caused amino acid change S534F.

**Table 1:** Mutations of directly sequenced influenza A (H7N9) virus and that obtained from chicken egg culture*

Gene	Position	Direct sequencing	Chicken egg culture	Amino acid change
PB2	18	A	G	Synonymous
PB2	1601	C	T	S534F
PB1	303	G	A	Synonymous
PB1	825	G	A	Synonymous
PB1	2274	A	G	Synonymous
PA	2115	G	T	Synonymous
PA	2119	G	T	V707F
PA	2127	A	C	Synonymous
NS	176	G	A	R59H in NS1
NS	792	C	T	P107L in NS2
NS	803	G	C	Synonymous
NS	804	T	A	Synonymous
NS	805	C	A	V111Q in NS2
NP	387	A	T	Synonymous
NP	438	T	C	Synonymous
NP	480	T	C	Synonymous
NP	648	A	G	Synonymous
NP	657	T	C	Synonymous
NP	663	A	G	Synonymous
NP	892	T	C	Synonymous
NP	962	G	A	S321N
NP	982	C	T	Synonymous
NP	1086	G	A	Synonymous
NP	1113	G	A	M371I
NP	1200	G	A	Synonymous
NP	1251	C	T	Synonymous
NP	1257	T	C	Synonymous
NP	1440	T	C	Synonymous
*PB, polymerase basic; PA, polymerase acidic; NS, nonstructural; NP, nucleocapsid protein.				

The influenza A (H7N9) genome also demonstrates significant intraspecimen heterogeneity. Deep sequencing revealed that the average coverage (ratio of the total number of nucleotides of all reads to the length of the reference gene) of the 8 genes was quite inhomogeneous. Average coverage (± SD) was highest for neuraminidase (NA) (131.94 ± 30.25) and second highest for NP (130.41± 27.01). The average coverages of PB2, PB1, PA, matrix protein, and hemagglutinin were 99.89 (± 22.49), 95.35 (± 21.34), 43.35 (± 14.13), 53.73 (± 17.67), and 69.82 (± 19.02), respectively. Average coverage was lowest for NS (27.73± 11.31). 

Besides the gene abundance, the genome sequence of influenza A (H7N9) virus also demonstrated heterogeneity (the heterozygous peak threshold 80%). In total, 22 positions were confirmed by PCR and Sanger sequencing to be heterogeneous (Table 2). In the NP segment, 4 positions demonstrated heterogeneity; 3 were synonymous and 1 induced amino acid change E421K. In the NS segment, 3 positions demonstrated heterogeneity; 2 were synonymous and 1 induced amino acid change R140W. In the hemagglutinin segment, 7 positions demonstrated heterogeneity; 6 were synonymous and 1 induced amino acid change H242Y. In NA, 3 positions demonstrated heterogeneity; 2 induced amino acid changes (S92L and S108L) and 1 was synonymous. In the PA segment, 2 positions demonstrated heterogeneity; both were synonymous. In the PB2 segment, 3 positions demonstrated heterogeneity; all were nonsynonymous (S532L, S533L, and S534F). All these heterogeneous sites were confirmed by PCR and Sanger sequencing; only 1 site overlapped with the mutation sites after passage in embryonated chicken eggs.

**Table 2:** Heterogeneous genomic positions of directly sequenced influenza A (H7N9) virus and its protein differences from other viruses*

Protein	Heterogeneity, nucleotide position in gene sequence: nucleotides)†	Amino acid position in protein sequence‡	Direct sequencing§	Culture§#	A/Anhui/1/2013§	Consensus of isolate from humans§**
HA	330: C>T	110	F	F	F	F
HA	360: C>T	120	L	L	L	L
HA	696: T>A	232	V	V	V	V
HA	724: C>T	242	H242Y	H	H	H
HA	762: C>T	254	F	F	F	F
HA	780: C>T	260	F	F	F	F
HA	1441: C>T	481	H	H	H	H
HA		65	M	M	R	R
M2		10	L	L	P	P
M2		24	D	D	E	E
NA	275: C>T	92	S92L	S	S	S
NA	323: C>T	108	S108L	S	S	S
NA	408: C>T	136	I	I	I	I
NA		40	S	S	G	G
NA		300	V	V	I	I
NA		340	I	I	N	N
NP		321	S	N	N	N
NP	858: C>T	286	A	A	A	A
NP	583: G>A	195	R	R	R	R
NP	1261: G = A	421	E421K	E	E	E
NP	1260: C = T	420	F	F	F	F
NP		371	M	I	M	M
NS2	546: C>>T	55	L	L	L	L
NS2	543: C>>T	54	D	D	D	D
NS1	418: C>>T	140	R140W	R	R	R
NS1		59	R	H	R	R
NS2		107	P	L	L	L
NS2		111	V	Q	Q	Q
PA	174: C>A	58	G	G	G	G
PA	1305:C>T	435	I	I	I	I
PA		618	K	K	T	T
PA		707	V	F	F	F
PB1		200	I	I	V	V
PB1		368	I	I	V	V
PB1		454	L	L	P	P
PB1		637	V	V	I	I
PB1-F2		42	C	C	Y	Y
PB1-F2		51	T	T	M	M
PB1-F2		70	G	E	G	G
PB1-F2		77	L	L	S	S
PB2		534	S	F	S	S
PB2		591	K	K	Q	Q
PB2		627	E	E	K	K

*HA, hemagglutinin; M, matrix; NA, neuraminidase; NP, nucleocapsid protein; NS, nonstructural; PA, polymerase acidic; PB, polymerase basic. †Position of first nucleotide = 1. ‡Position of first amino acid = 1. §Types of amino acids. #Virus cultured in chicken eggs. **Thirteen influenza A (H7N9) viruses isolated from humans; data from Global Initiative on Sharing All Influenza Data.

Compared with the reference influenza A (H7N9) virus strain A/Anhui/1/2013, the influenza A (H7N9) virus demonstrated prominent sequence differences (Table 2). In particular, the amino acid at the 627 position of PB2 of A/Anhui/1/2013 is K, whereas the corresponding amino acid in the subtype H7N9 genome is E. The amino acid at the 368 position of PB1 of A/Anhui/1/2013 is V, whereas the corresponding amino acid in the subtype H7N9 genome is I. The E627K mutation in PB2 and the I368V mutation in PB1 are closely associated with the virulence and transmissibility of avian influenza A virus in mammals (*1*). E627K in PB2 was observed in A/Shanghai/1/2013, A/Shanghai/2/2013, and A/Anhui/1/2013 viruses (*1*). A/Zhejiang/DTID-ZJU01/2013 virus does not have this mutation but has a complementary mutation D701N in PB2 (*2*). I368V in PB1 was observed in A/Shanghai/2/2013 and A/Anhui/1/2013 viruses, but A/Shanghai/1/2013 virus does not have this mutation (*1*).

MEGA5.0 (www.megasoftware.net) was used to construct the phylogenetic trees on the basis of the nucleotide sequences of all influenza A (H7N9) viruses in the Global Initiative on Sharing All Influenza Data (GISAID) database (*7*). We conducted 2 rounds of phylogenetic analysis. First, to examine whether this subtype H7N9 virus is clustered with the available subtype H7N9 strains, we included all influenza A (H7N9) viruses in the GISAID database. To construct the multiple sequence alignment, we used the MUSCLE package with default parameters (www.megasoftware.net/); then, to construct the phylogenetic trees with 1,000 bootstrap replicates, we used the minimum-evolution method. Results suggested that all 8 genome segments are closely related to the available influenza A (H7N9) virus strains. 

**Figure 1:**
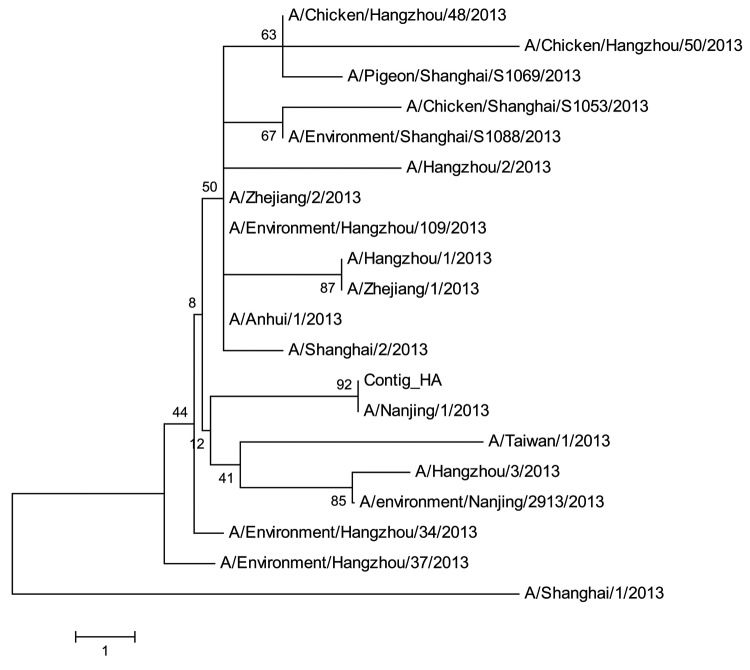
Phylogenetic tree of the influenza A (H7N9) viruses isolated in China in 2013, based on the hemagglutinin gene segment. Scale bar indicates nucleotide differences per unit length.

**Figure 2:**
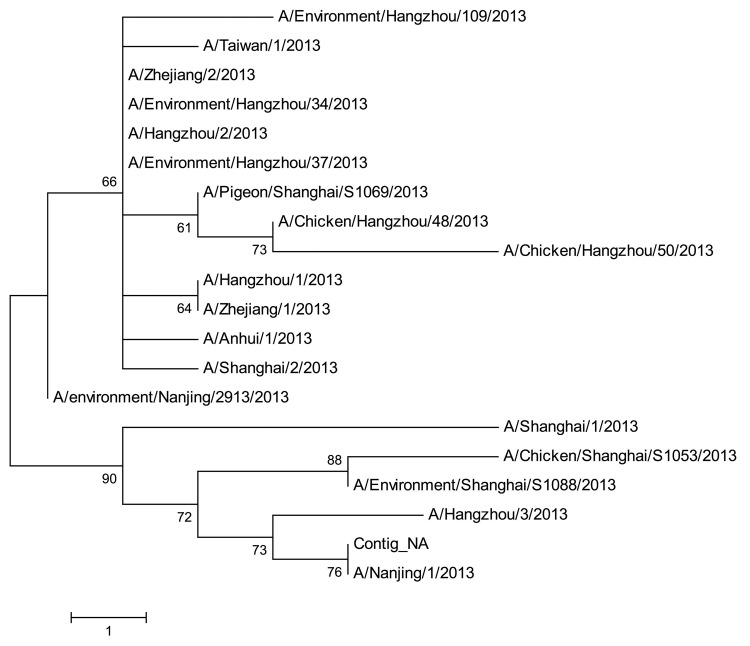
Phylogenetic tree of the influenza A (H7N9) viruses isolated in China in 2013, based on the neuraminidase gene segment. Scale bar indicates nucleotide differences per unit length.

We next included all influenza A (H7N9) viruses isolated in China in 2013 to closely investigate the relationships between this virus and available subtype H7N9 genomes isolated during epidemics. However, the phylogenetic topologies based on different gene segments were not consistent (Figures 1, 2; Technical Appendix Figures 1–6), suggesting that the influenza A (H7N9) virus may have persistently evolved for a while (*8*).

## Conclusion

Using deep sequencing technologies, we derived the full-length genome of the novel influenza A (H7N9) virus directly from the sputum specimen of a patient, without conducting virus culture. The full genome revealed substantial sequence heterogeneity within the specimen, obvious sequence variations from that obtained from embryonated chicken eggs, and prominent differences from the available influenza A (H7N9) strains, most of which were sequenced after culture.

Technical Appendix Phylogenetic trees of the influenza A (H7N9) viruses isolated in China in 2013, based on gene segments.
