# Supplementary material for: Full Genome of Influenza A (H7N9) Virus Derived by Direct Sequencing without Culture
Source: Emerg Infect Dis. 2013 Nov;19(11):1881–4. doi: 10.3201/eid1911.130664 (PMC3837655; doi:10.3201/eid1911.130664)
Supplement: Technical Appendix — Phylogenetic trees of the influenza A (H7N9) viruses isolated in China in 2013, based on gene segments. [file 13-0664-Techapp-s1.pdf]

Article DOI: http://dx.doi.org/10.3201/eid1911.130664

Full Genome of Influenza A (H7N9) Virus Derived by Direct Sequencing without Culture

Technical Appendix

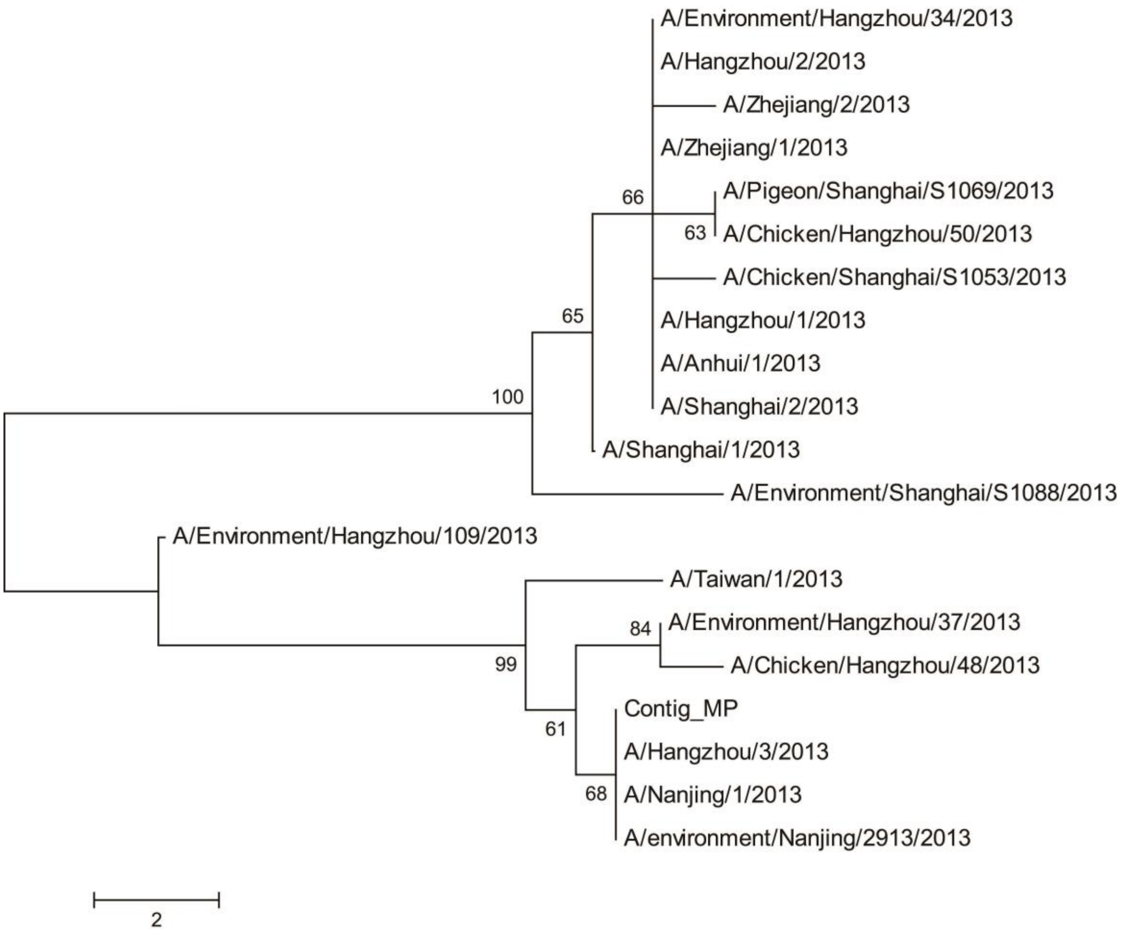

Technical Appendix Figure 1. Phylogenetic tree of the influenza A (H7N9) viruses isolated in China in 2013 based on the matrix protein gene segment. Scale bar indicates nucleotide differences per unit length.

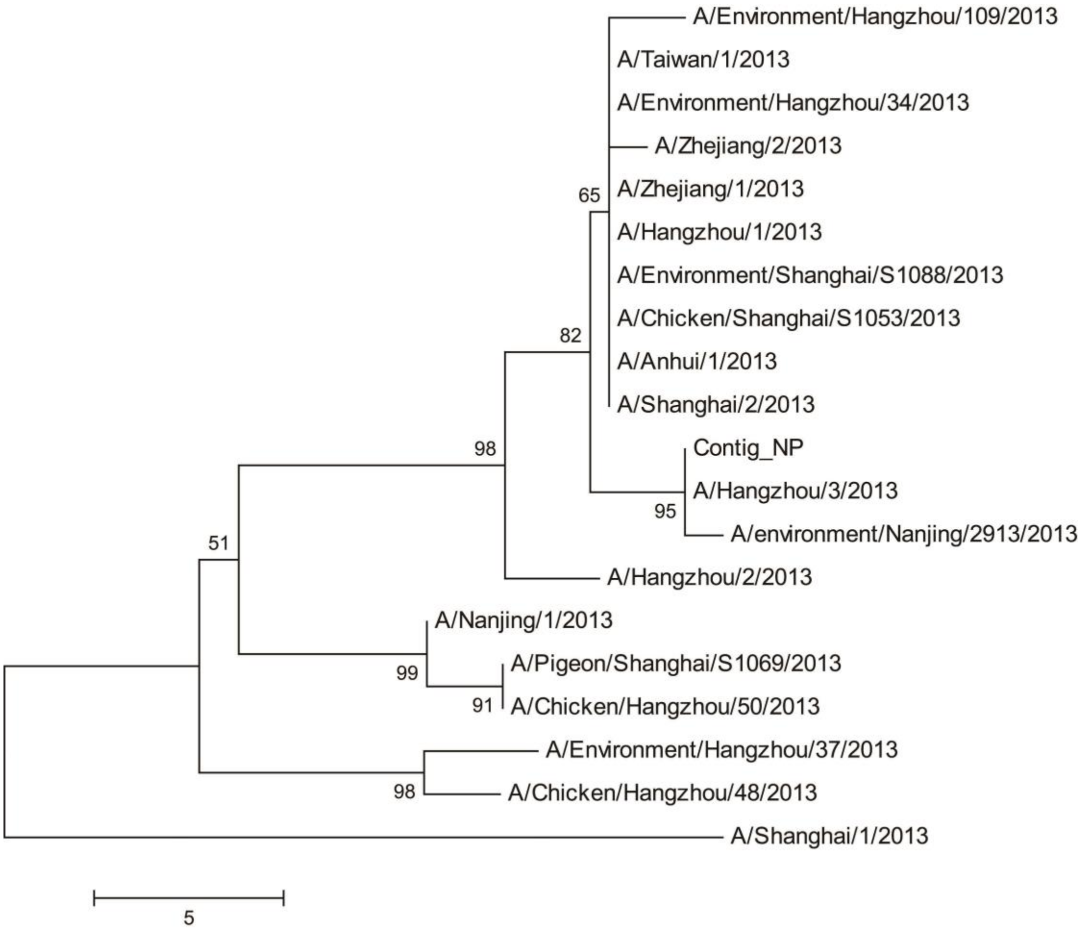

Technical Appendix Figure 2. Phylogenetic tree of the influenza A (H7N9) viruses isolated in China in 2013 based on the nucleocapsid protein gene segment. Scale bar indicates nucleotide differences per unit length.

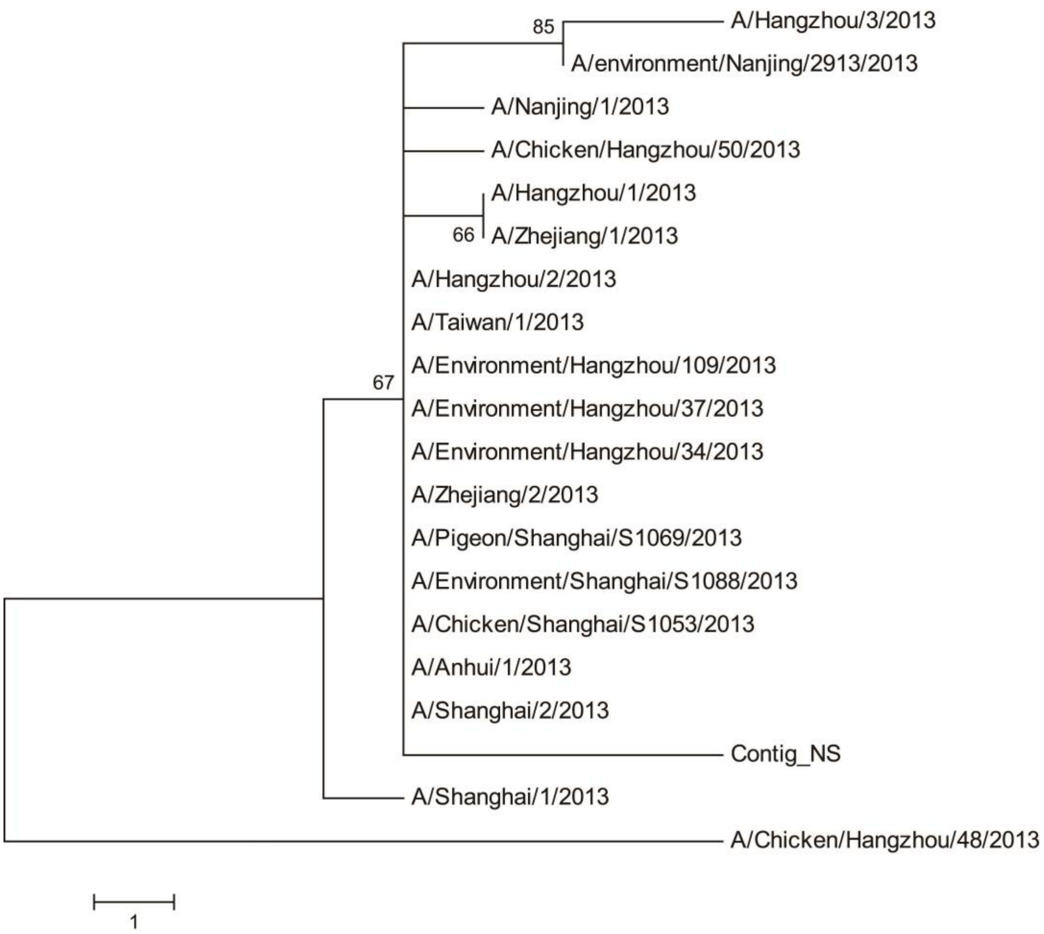

Technical Appendix Figure 3. Phylogenetic tree of the influenza A (H7N9) viruses isolated in China in 2013 based on the nonstructural protein gene segment. Scale bar indicates nucleotide differences per unit length.

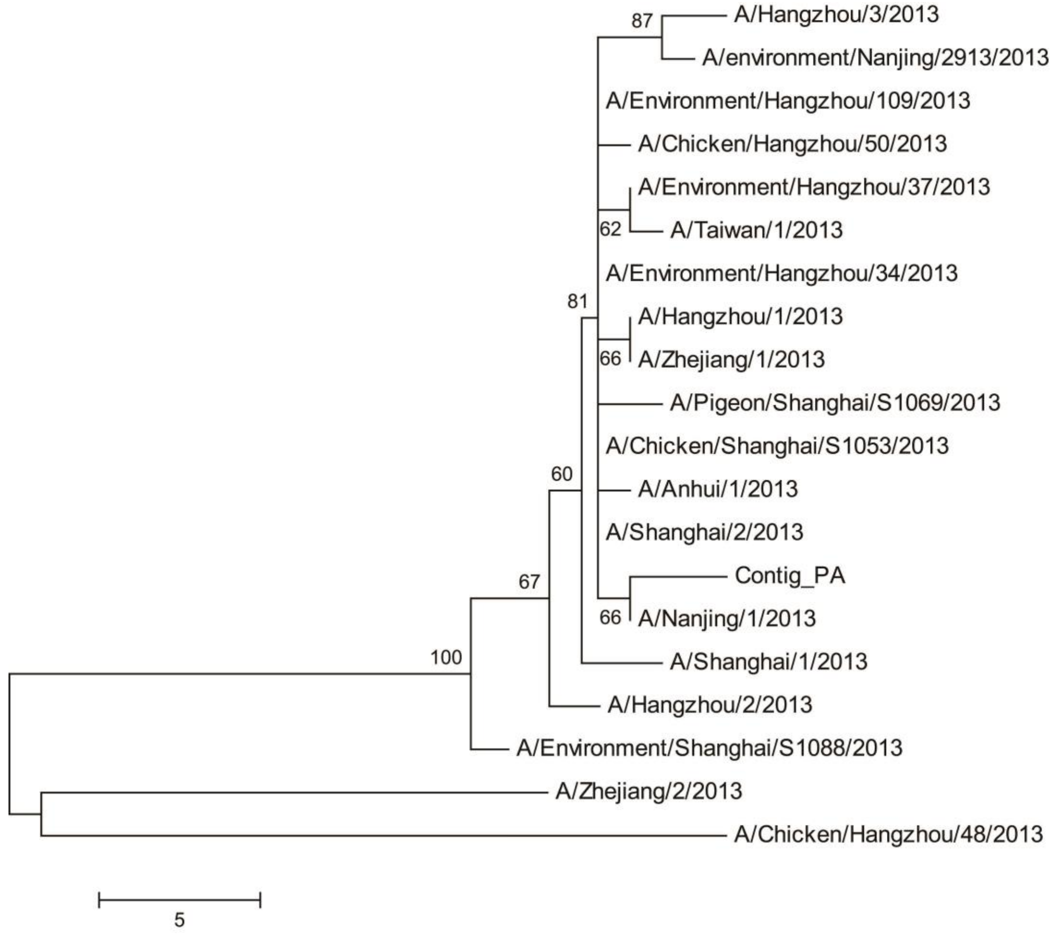

Technical Appendix Figure 4. Phylogenetic tree of the influenza A (H7N9) viruses isolated in China in 2013 based on the polymerase acidic protein gene segment. Scale bar indicates nucleotide differences per unit length.

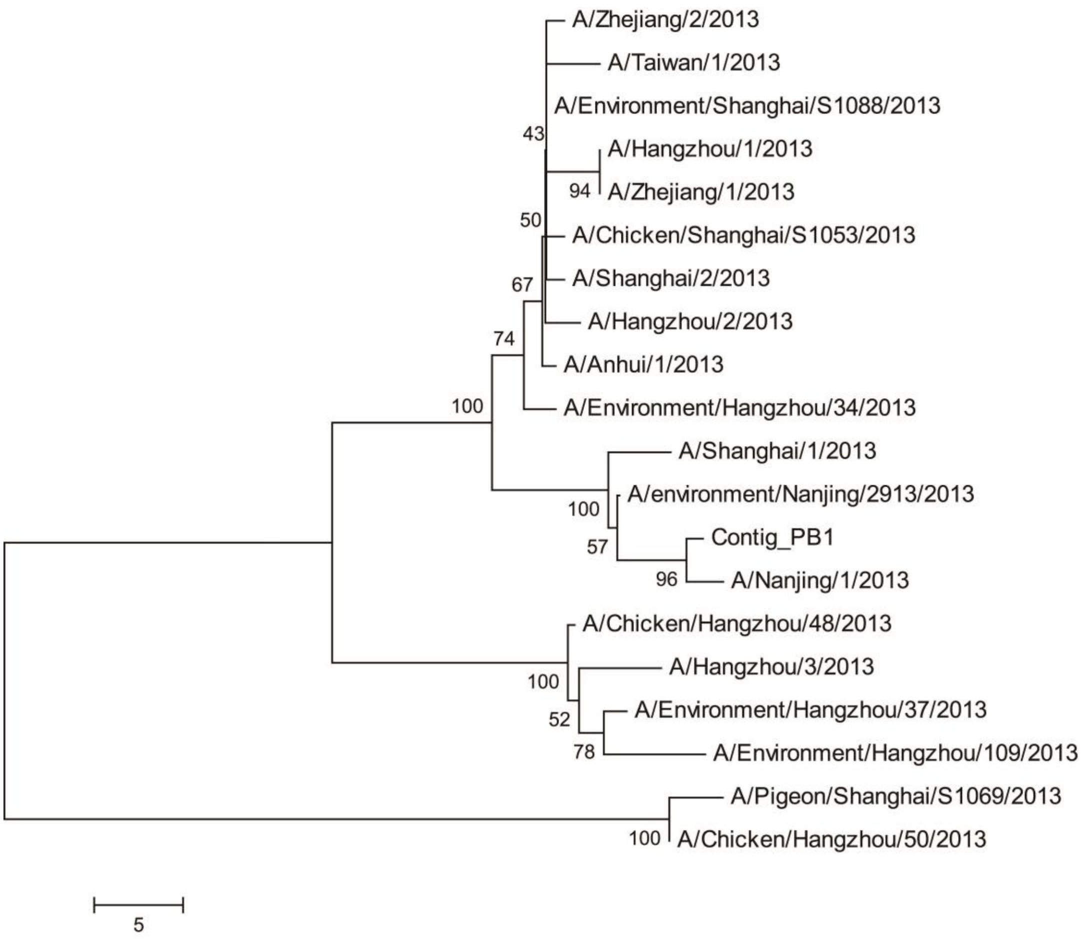

Technical Appendix Figure 5. Phylogenetic tree of influenza A (H7N9) viruses isolated in China in 2013 based on the polymerase basic protein 1 gene segment. Scale bar indicates nucleotide differences per unit length.

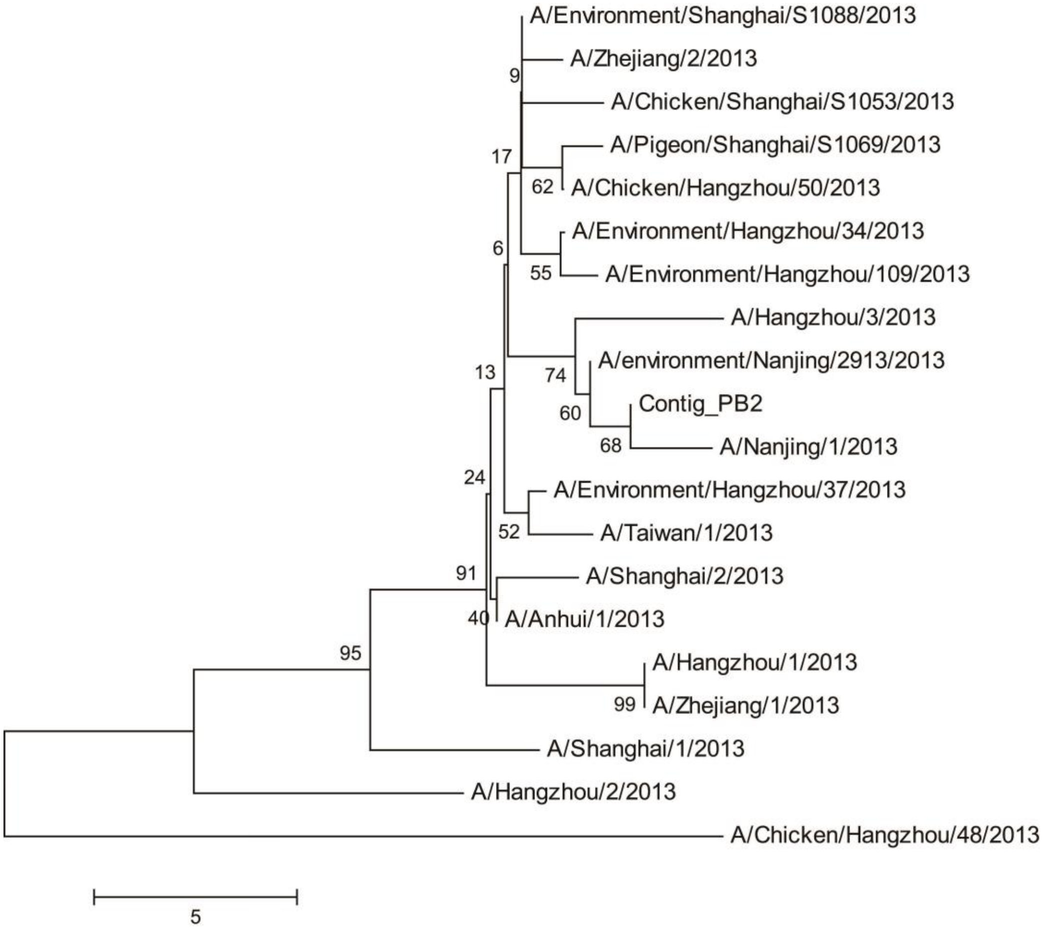

Technical Appendix Figure 6. Phylogenetic tree of the influenza A (H7N9) viruses isolated in China in 2013 based on the polymerase basic protein 2 gene segment. Scale bar indicates nucleotide differences per unit length.
